# Supplementary material for: Co-development of an acceptance and commitment therapy-based intervention to increase intrinsic motivation of adolescents to change weight: The AIM2Change study
Source: PLoS One. 2025 Nov 3;20(11):e0308770. doi: 10.1371/journal.pone.0308770 (PMC12582442; doi:10.1371/journal.pone.0308770)
Supplement: S1 Table — (DOCX) [file pone.0308770.s002.docx]

Table of changes

The table of changes is an iterative document that has been contributed to following PPI meetings and updated throughout the iterative development phase of the intervention.

Coding key:

IMP = Important behaviour change  
EAU = Easy and uncontroversial 
REP = Repeatedly 
EXP = Experience (specify PPI, experts, literature) 
NCON = Does not contradict experience or the guiding principles  
NC = not changed (give a reason)

MoScoW = Must do, Should do, Could do, Would like to do

# Pre-Trial PPI feedback

Note: this has already been included in the initial intervention development paper (24)

| Aspect of the intervention | Negative Comments | Positive comments | Possible changes | Reason for change code | Agreed change | MoScoW  (Must do, Should do, Could do, Would like to do) |
| --- | --- | --- | --- | --- | --- | --- |
| Conceptual and theoretical factors | | | | | | |
| Novel approach |  | The approach was novel and enabled them to think about their feelings in a way they had not before. |  |  |  |  |
| Internal/self-motivation | PPI group (PPAG) members described repeated failed attempts at weight loss until they themselves felt they were ready. The challenge is that the YPs have been referred to the programme; we need to tap into a way of getting them ready to start, from a place where they may not have chosen to be referred. | Thought to be important for successful weight change. Not going to continue with an intervention that *“makes them feel rubbish”.*  GB: “*Got to have that moment where you think I need to do this for me”* and GT: agreed need to *“decide for myself”* to make the change and to stop thinking of healthy eating as a negative thing to be doing, instead framing it as a positive change. | Consider methods to drive internal motivation including visualisation, clarification of values.  Reduction of external pressures may give space for internal motivations. | IMP, EXP | Incorporate methods to drive internal motivation including visualisation, clarification of values. | Must do |
| Holistic Approach |  | This concept came up in both adult PPI sessions – the importance of seeing the whole person rather than purely focussing on what they eat. BC: towards end felt the approach was “*empowering, hopeful and for the whole person”*  The lack of focus on specific food, and more about whole person and behaviour as a strong benefit. | Option to completely remove mentions of food, eating and body image or to retain the current balance which connects with these topics in addition to a whole person approach. | NC | The team felt the purpose of this programme was specifically to address the big challenges young people were experiencing with eating, weight and body image, rather than making this a generic wellbeing DNA-V programme (which already is in existence). Therefore, a focus on this area was maintained whilst also allowing young people to bring broader issues to the therapy. |  |
| Flexible approach as opposed to strict rules. | For those with poor nutritional knowledge, more guidance may be required to establish a healthy diet. | PPI members reflected on their experience of wanting something more because it had been restricted- a flexible approach is preferred. |  | Exp. NCON | As the clinic already offers nutritional education, we hope that patients have learnt during their initial 6-months at the COCO clinic. Dietary guidelines and calorie counting is also offered so this intervention seeks to take a different approach to support those for whom that approach has not worked. |  |
| Individualised approach | The importance of making the intervention relevant to the individual's interests and motivations (gave example of his therapist using his interest in cars) |  | Develop individual rapport with patients enabling metaphors and examples to be tailored to their needs | EXP | Therapists’ confidence with the programme material will need to be considered when thinking about their ability to adapt interventions on the spot.  If possible, this would be the ideal, but feasibility will need to be reviewed. | Would like to do |
| Format and structure of sessions | | | | | | |
| 1-2-1 sessions |  | Felt that they were more comfortable sharing 1-2-1 as it offered greater opportunity to be candid.  Ppt.10, Session 1*: “ I like it better just me”.*  AS: *“Why do you say that”?*  Ppt.10: *“I can just say most things”.*  AS: *“So you can be more open and honest”?*  Ppt 10: *“Yes”.* | Run optional groups, run just 1-2-1 | NC | Sessions will stay as 1-2-1 sessions at the moment, to enable the tailored approach and therapy-patient relationships that has also been raised. | Must do |
| Where to hold the sessions |  | As much choice given to the young people as possible.  Ppt7, Session 1: *“ I think it is just as good online as it would be face to face, to be honest. I don’t think it would make a difference whether it is online or face to face to be honest. As it went on I felt better and better about it as it was so good”.* | In-person sessions to be offered exclusively, optional in-person sessions to be offered, only online sessions | NC | Young people maintaining the choice about where they would receive the therapy, with online being the main option unless they do not have a private place to work. | Must do |
| Parents present in the sessions. | Young people may struggle to discuss how they feel in front of parents, especially before they have built rapport with the clinical team enough to feel safe to discuss their feelings.  Holding these discussions in front of family may be particularly sensitive if the young person feels their upbringing and/or home food environment has contributed to their weight problem.    Equally parents may have questions and experiences they want to share with the clinicians but not in front of the young person. | Some parents had been present in initial sessions with participants but had realised that their presence may compromise participant engagement and openness in sessions.  Mother / Participant 8: *“I was* *actually with* *him and he became quite mute and not wanting to discuss anything about it, any situation [that] was going on. But I suppose when he’s done the counselling (with JC) it was a lot better for him and in that he could, I would leave him to* *it and I wasn't present”.* | Splitting sessions so part is done with the YP alone to develop trust and confidence to share with their PG  Or  Offering the young person the chance to invite their parent to join or not | REP, EXP, NCON | The young person will get the chance to invite their parent when they want to - session topics will be given a week ahead so young people can make a session-by-session choice.  Currently, no session time has been allocated for parents-only, however this will be reviewed. | Should do |
| Default of inviting the parent to support | The parent may not be the most appropriate support, in some cases a grandparent or other figure may be the child’s key support. | As much choice given to the young person about the session is a good thing. | Young people being given the option to bring an alternative support person, not defaulting to always being the parents. | EXP, NCON | This option will be offered to participants and continually reviewed.  No support person other than a parent was present in subsequent sessions | Could do |
| Online (real-time) as the platform for therapy | Might create problems if no privacy at home without parents /siblings. Don’t know who else is there to “*make sure they are saying the right things”*.  Example AS: *What about your situation at home is it private enough for you?*  Ppt 9: *Yeah.*  Ppt 9: *Go away* (to brother!)_ | Some cases Zoom better, eg video off, may help to be relaxed in a comfortable place, more honest.    Ppt 7: *I like doing it on Zoom and I felt quite safe and secure.*  Mother: *He was in his room.*  *He was on his territory.* | Check with participant if privacy an issue prior to starting session and discuss potential solutions with parents if necessary | EXP (Participant) | To be continually reviewed at each therapy session | Must do |
| Terminology | | | | | | |
| Terminology – unhooking |  | The wording resonated – in particular ‘unhooking’ and ‘hooked’ was described to explain how they feel about being absorbed by darker thoughts, with strategies such as going for a walk being ‘unhooking’ providing an opportunity for reflection | Offer several activities to try for each session as individual differences in how each activity will be viewed and received. | EXP (Participant) | To be continually reviewed at each therapy session | Must do |
| The use of the term ‘Choice’ | Does choice have connotations of fault and blame? That ‘choice’ is a difficult word. As YP often told they have choices but often they are already made (by adults, socioeconomic position, genetics - CG). HH “*YP may not yet understand that they are in control of their behaviour.”* That they might not realise they are making choices. JW – remembered feeling that she didn’t understand her emotions “never mind that I had a choice”. Linked to DF (Thurs11th) who felt when muddled, she didn’t feel she had a choice in the way she behaved around food. |  | think about how to use the word choice so it avoids negative feelings surrounding not having made good ones previously. Raising awareness of the choices we make all the time – some YP may not be aware or even able to make ‘good’ choices when feeling emotional and pressured. |  | Work to be done on explaining the concepts, before then introducing choice as something we would work on giving the YP. Rather than implying that they have always had choice and have been taking the wrong choice up until this point. |  |
| Using the term ‘mindfulness’ | The term ‘mindfulness’ is overused especially during school and when stress is discussed. It makes people stop listening as it feels like more of the same. | Some adolescent PPI members had positive experiences and a regular mindfulness practice, therefore connecting that the activities were mindfulness allowed them to build on their skills  Important to not underestimate young people's interest and engagement: | Using the term ‘awareness’ i.e. bringing your awareness to certain experiences, rather than mindfulness may help tap into the beneficial effects of mindfulness without the cliché preconceptions. | EAU, REP, EXP NCON | Both terms will be used and the preferred language will be discussed with each young person. This is an area for continued monitoring and can be changed if there is a clear pattern. | Could do |
| Specific activities | | | | | | |
| Three mountain metaphor | The metaphor simplifies the challenges of the journey and the external pressures including peer pressure and technology that impact the journey  One person felt it could be viewed competitively, so suggested making sure it wasn’t viewed as a race or competition | The collaborative aspects of the therapeutic relationship are clear, the approach of tackling these problems together, with lots of emphasis on how the young person is involved in shaping this process.  It was felt this approach was “inviting”.  “Invitation to come on a joint journey”  “makes them know it’s what they want rather than being told to do something.” | Ensure that the diagram of the mountain includes notable ‘up’ and downs’ and discuss them in the explanation of the metaphor | EAU, EXP, NCON | Yes | Should do |
| Lottery | YP’s weren’t as keen on the lottery example as made them feel selfish. Found it hard to decide how to see yourself. |  | Continue with the exercise with greater explanation and space to explore, change activity for another that explores values | EXP | Change the activity, as the activity was one of many ways in which to explore values, an activity with less explanation is required on a time-limited programme | Should do |
| Inflatable ball activity |  | The activity facilitated conversation; it was easier to discuss having done the activity together.  People then built on the metaphor, continuing to use it in new ways to explain how they felt.  *“Feel like you described my teenage years” “a new way to consider this”.*  Strength in that the conversation evolved, with people still using the metaphor to explain how they felt in other situations | Continue with the metaphor, remove the metaphor. | NC | Metaphor is maintained as it facilitated useful conversations and understanding. | Must do |
| The choice point video | The video simplifies the decision to make the life-enhancing decision, rather than the habitual non-healthful behaviour.  Video features a very slim woman with protruding collarbones with PPI members found drew their attention.  Video only features a woman.  Some of the language/issues were not necessarily the right focus for YP (e.g. financial worries). | The video captured attention and offered a concise snapshot of what was perceived as an interesting and novel approach. Left people wanting to know more about the approach.  The video offered hope that there was a solution not just to their weight but to how they were feeling more broadly. Gave them a sense that they didn’t need to feel stuck and left them feeling more positive.  Discussions were held about how the video was targeted at adults, but that this wasn’t necessarily a negative thing as for many YP’s it would be empowering to know that they were being treated as adults.  “*Got the point across without being super cringy”.* | Caveat the video with the understanding that this is a simplified version of what is actually going on.  There are two versions of the video, so ensuring the version with the slim woman is not used. | Exp. NCON |  | Should do |
| Visualising your mind as a character | Not all people enjoyed trying to draw and visualise their minds. One person who was less keen on the activity felt their mind often encouraged them to engage in the health beneficial behaviours. | Some people found the idea that our mind tells us the same repeated stories useful and enjoyed reflecting on this.    Members explained battling with the two sides of their mind | Assessing where the YP is at individually may help to understand whether this is a useful activity for them | EXP | Developing the baseline that these are all things to experiment with/try. Some will feel helpful, others may not, and that is okay – we can just keep working with/building on the things that feel they work for that individual. Not everything will work for everyone. | Could do |

# PPI feedback during the study

Coding key:

IMP = Important behaviour change  
EAU = Easy and uncontroversial 
REP = Repeatedly 
EXP = Experience (specify PPI, experts, literature) 
NCON = Does not contradict experience or the guiding principles  
NC = not changed (give a reason)

MoScoW = Must do, Should do, Could do, Would like to do

| Aspect of the intervention | Negative Comments | Positive comments | Possible changes | Reason for change code | Agreed change | MoScoW  (Must do, Should do, Could do, Would like to do) |
| --- | --- | --- | --- | --- | --- | --- |
|  | | | | | | |
| Participant Information Sheet (PIS) | Black writing on white background is hard for people with dyslexia to read and comprehend. Suggest yellow or green background, black text.  Should we also consider those who are colour-blind. |  | A modified version of the PIS could be sent (by post or email) as soon as possible after establishing that they struggle with black on white, rather than make all the PIS yellow or green. | EXP (PPI), EAU | Modified version of PIS to be sent out to those who need it. Colours used are largely blue on white, so people who are red/green colour blind should be able to see these without further changes. | Could do |
| PIS | At least four attendees of the session commented that the PIS contained too much information, with too much text. |  | Increase font size, break up the sections of text, use more pictures to describe the study. | EXP (PPI), EAU | All changes made to final version of PIS | Must do |
| PIS | Parents in the group voiced that they wanted time to consider the information in the PIS, either by suggested the PIS is sent to them in advance or to be allowed time to consider after being given it at clinic. |  | (i) Could send PIS out in advance for parents and YP to read; (ii) could give the PIS out at clinic for people to take away to consider; | EXP (PPI adults), EAU, NC | No change needed as plan was for the project to be introduced to YP and their parents during clinic by their treating clinician and given PIS to take away to read. Decisions regarding participation will not be rushed. |  |
| PIS | Have the fact that that the sessions will be individual and one-to-one, not group sessions, near the top of the information. |  | Ensure this information is given prominence in the PIS | EXP (PPI adults), EAU | PIS amended accordingly | Must do |
| Parent attendance at therapy sessions and consensus meeting | Those present at the PPAG meeting agreed that YP might not be able to speak as freely in the session with the parent there. | Several voices agreed (if not all) that the YP should be allowed to choose whether parents could join the therapy sessions and the consensus meeting with the YP | The choice of who attends the therapy sessions and consensus meeting could be given to the YP, parent or a joint decision | EXP (PPI adults and YP), EAU | Agreed to allow the YP to decide who should attend each session. | Must do |
| Support sheets with summary of session content |  | Several voices agreed that ‘parent/carer support’ sheets for each session would be welcomed and very helpful so that they could support their YP. One person said that this would be essential for them. | (i) prepare general support sheets per session to send to parents afterwards; (ii) prepare bespoke sheets for each session; (iii) keep focus of intervention on the YP and allow them to choose what to share with parents. | EXP (PPI adults), NC | Decided not to offer parent support sheets during this initial co-development study, as the sessions were iteratively changing during the process and sessions were often quite bespoke to each individual (albeit following the session plan where possible). Resources did not allow a summary for each session to be prepared. A point to consider for future work. | Could do |
| Name of the PPI group |  | “*I like that it is an advisory group it's not too pushy”*  *“Happy with the term advisory group.”* | Offered various names of the group including Patient & public advisory group, Young persons ‘advisory group, Young ambassadors. | EXP (PPI adults) | The feedback from attendees suggested that PPAG was the most popular name for referring to the PPI group during the study. | Should do |
| Content of sessions | Crucial to the YP that the sessions are not just about weight, as the sessions could be overwhelming if all about weight-loss. | SHINE group also liked the idea of the primary focus being on health and wellbeing rather than weight loss alone | Allow opportunity for YP to bring up other issues during their sessions. | EXP (PPI YP) | During rapport building stage and early sessions, YP given opportunity to talk about their important issues. Therapist to bring in eating and weight management when appropriate | Must do |
| Length of sessions |  | “*I think that one hour a week is a good amount as you can have a week to reflect and possibly start to change their habits for the next week”* | Gave options of 30, 45 and 60 minute length sessions | EXP (PPI YP) | Decided to keep sessions to 45 minutes, with 15 mins for the interview at the end, as might be harder to maintain concentration online for any longer period | Must do |
| Intro video | Only should use the introductory video to provide context for more detailed discussion about each of the DNA-V characters.  Considered to be a bit short,and didn’t mention all the struggles people living with overweight experience. | “*I feel like the video is good because it is easily understandable for young people which will help them with the whole project”* | Options include keeping in the intro video or the therapist to provide the introduction to the DNA-V model verbally. | EXP (PPI YP) | Decided to keep in the introductory video as some found it valuable but to add some explanation by the therapist to ensure the YP understood the concepts. | Should do |
| Language used | “*I think possibly only having the word overweight would be better rather than obesity. Obesity seems more of a strong word if you know what I mean so it might put people off.”*  YP were concerned about the stigma associated with the word obesity | “*Also only saying overweight may bring in a bigger range of people and sizes.”*  ‘Overweight’ was thought to be better than the term ‘excess weight’. | Could discuss the words to be used in the therapy with each individual / ask for favoured words and this would serve as a ‘trust exercise’ with the therapist so more comfortable with each other | EXP (PPI YP) | Therapist used general terms such as ‘weight issues’, ‘body image’, and avoided using words such as ‘obesity’ and ‘overweight’. | Could do |
| Delivery of sessions | YP concerned that if held online they might not be able to find a place for a confidential chat | Others liked the idea of not having to travel and independently joining the sessions | Online or in person | EXP (PPI YP) | Decided on balance to plan for the therapy sessions to be online | Should do |
| Contact with YP | Email not the best form of communication with YP | Suggested asking permission to use YP mobile phone numbers to communicate on WhatsApp or text messages | Ask for preferred contact details when given PIS | EXP (PPI YP) | The study paid for access to a mobile phone so that the therapist could contact YP and parents for reminders and could receive messages close to session times | Must do |
| Planning feasibility, proof of concept trial | Some people might not think it was worthwhile if ended up in the control group. | Thought it would still be worthwhile (insights into research, experience of being in a trial, giving back to other YP) if we made it clear they would be helping. Suggested we could offer them the therapy after the initial randomised period. | Include a control group and make possible benefits clear, or allow control group to access the therapy at the end of the initial period | EXP (PPI YP) | Short period of planned grant application does not allow sufficient time for the control group to access the therapy. | Could do |
| Questionnaires for feasibility, proof of concept trial | PedsQL – not sufficiently tailored to weight issues, narrow response options.  AAQ-W – negatively worded, would have liked to see more positive items. Too centred on the self rather than comparison with others. Might make some feel uncomfortable so would need explaining.  DEBQ – language not clear, formal and old-fashioned (e.g. ‘seldom’, ‘desire to eat’) | OK to complete these sorts of questionnaires (e.g. AAQ-W) if the YP understood who would see their answers and if they didn’t have to add their name.  Liked that the DEBQ was more specific to the main topic (eating).  YP preferred the TFEQ questions and response options. | Could include the various questionnaires or not | EXP (PPI YP) | Decided to include the PedsQL as a measure of quality of life, and TFEQ as a measure of eating behaviour | Should do |

# Participant feedback during the study (Co-development process)

Coding key:

IMP = Important behaviour change  
EAU = Easy and uncontroversial 
REP = Repeatedly 
EXP = Experience (specify PPI, experts, literature) 
NCON = Does not contradict experience or the guiding principles  
NC = not changed (give a reason)

MoScoW = Must do, Should do, Could do, Would like to do

| Aspect of the intervention | Negative Comments | Positive comments | Possible changes | Reason for change code | Agreed change | MoScoW  (Must do, Should do, Could do, Would like to do) |
| --- | --- | --- | --- | --- | --- | --- |
| Developing interview techniques | | | | | | |
| More targeted questions | Researcher / Interviewer AS: Finding it difficult to pinpoint activities during interviews to gain in-depth feedback |  | Using a scoring system at the end of each activity, to focus YP on the research side of the sessions and support AS in his questioning, suggested by ClinPsych CS | EXP participant | Implemented scoring system where the Ppt self-reports on a scale 1-10 usefulness of strategy. This was first implemented with Ppt 4 in session 3. | Must do |
| Not feeling comfortable discussing therapy with another person | Participant who found it difficult to be open in the sessions and requested to speak to therapist rather than interviewer regarding co-development aspects of the therapy  JC: *“Is it difficult like him (AS) just joining at the end”?*  Ppt3: *“Yeah”.*  JC: *“If it was a different, somebody different to work with like a lady, would that be better”?*  Ppt3: *“Yeah”.* | JC: “*So instead of speaking to AS it's fine that doesn't work out for you. Is there anything that you want to share? What was particularly helpful or that you wanna do more of or* *You wanted to do less of”?.* Example Ppt3: “*It’s the exercises I really like”*  JC: Yeah. What was your favourite one today Ppt3: It was the writing one  JC: “*Yeah, and it's a good way of getting stuff out. You know, if you really worrying about something, you don't have to share anyone, you can rip it all up and put it in the bin straight after. But sometimes it just helps you get it out of your head, doesn't it.*” | Option 1: The female therapist incorporated the ‘Think aloud’ items into the therapy session.  Option 2: The therapist asks the ‘Think aloud’ as a stand-alone interview post therapy | EXP (participant)  NCON | Therapist adopted changes in interview process in accordance with participant preferences and focus on the content of therapy session | Must do |
| Iterative changes to interview schedule |  | Consistent with reflective practice and as a team discussed the feedback from sessions and identified a need to amend the interview schedule for each session  Th interviews schedules were drafted to reflect the theme of the session; Advisor, Discoverer and Noticer etc and which specific strategies were used to support the focus of the session.  The topic guides also reflected more practical aspects of the sessions such as resource materials and structure/format of sessions, for example: | Add additional questions to schedule | EXP (research team) | Changes in interview schedule were made to reflect changes in the content of the therapy.  These were made on an iterative basis and specific to each session. Example: - AS: would you like to see more activities like those included in the therapy sessions?  AS: Would you like a workbook / resource? Would you like this at the beginning to work through, or materials sent after every session? | Must do |
| Session revision | | | | | | |
| Having a focus on eating / weight / self-image throughout | Ppt 2: “*There hasn’t been a lot of sort of tying in with eating”* | Ppt 2: *“I just wanna talk about stuff going on, it's really helpful.* (Not focus on weight)”.  JC: Do you think that will help what is going on in the CoCO clinic?  Ppt 2: *“Yeah, I think I will be more focused on my goals”* | Option 1. Changing to have a strong eating focus throughout  Option 2. Changing to prompt YP using more eating/body related examples throughout  Option 3. Continue to leave early sessions open to optional discussion | EXP | Because of the nature of the outcome measures being desired by clinicians and included in future research trials, it felt pertinent to ensure YP’s were given enough time and space to consider eating/weight/body image specifically during the sessions.  A decision was made to increase the prompts in early sessions, and if necessary, focus on these issues earlier in the programme. | Must do |
| AAQW |  | Scoping review by team highlighted measures of ACT related outcomes | Inclusion in therapy session or not | NC | Not included in sessions as didn’t feel fitting with co-development of therapy | Could do |
| Inclusion of DNA-V session planning tool |  | Training with DNA-V expert suggested benefits for allowing participant choice for order of sessions | Include tool in one of the early sessions | EXP (Expert) | Included tool in session? |  |
| General feedback on content | | | | | | |
| To have less reliance on the videos, especially the main introductory video with Ppts valuing the more tailored explanations | AS: *can you remember what those initials (DNA) stand for?*  Ppt 12: *No, she showed it to me, but she didn't like*  *properly explain it she just like briefly talked about it.* | Ppt 1: “*I feel like there should be more videos and more tasks”.*  Ppt 9: “I *really learned a lot actually... she was explaining to me. She (*JC) *explained it more better* (than the video)*”* | Not include videos and rely on verbal explanation. OR. Include videos but decisions to show should be made on a participant engagement basis with additional and tailored explanations where required | EXP (Participants)  NCON | Agreed to include videos and offer verbal explanation led by participant preference on a topic / strategy basis. | Must do |
| The focus on eating behaviour | Needed to be at an earlier time point / integrated throughout therapy sessions and linked to emotional wellbeing | Ppt 7: *It's something I wanted to talk about anyway... It's just good how much (food talk) there is, but I think it should sort of be mixed in with everything else at the same time. So it's constantly there, it's constantly implemented.*  *Yeah, it's an important thing to talk about. So having it there, whether it's a small aspect or big, it's important to be there, you know.* | Each session teaches a skill and connects to eating / body image | Agreed that therapist would gauge participant preferences for inclusion of food/eating behaviour at start of therapy sessions | EXP (Participants) | Must do |
| Physical copy of manual content for each session or at the beginning of session series? | AS: “*Do you feel like there would be any benefit from having a workbook to work alongside the sessions”?*  Ppt 12:  *“No, not at all.* | Ppt 7: “*I think it would be useful, but I don't think it should be something that everyone has to have. So, you can choose whether you have it, whether it's useful or not”.*  Ppt 12:   *“The workbook which is like homework. Nobody likes it...*  *AS: Is it too much like homework? Is that what you mean? Ppt 12: Yeah, it feels like homework”.*  Ppt 1: “*I think a workbook could be good in terms of like putting in a summary of what each session is about or putting in resources from each session into that workbook”.* | Considered supporting documents prior to session but as session content was responsive to participant needs in session documents could not be prepared *a priori.*  Documents could be provided post session but there was not a clear consensus among participants as to whether this was a useful approach? | EXP (Participant) | Raised in course of delivering sessions to all participants but will be considered for future trial of therapy | Could do |
| The wellbeing focus of psychology / AIM2Change was in contrast with CoCO/CEW main approach | Ppt 2: *“I mean the fact that you're at the CoCo clinic and told to kind of count calories and look at the diet and, you know, really focus on the food and take the drugs and so on with the very clear focus about, you know, losing weight and focusing on that. And then in the counselling (Aim2Change), it's much more about feeling good about yourself being positive and it's those two things are almost in conflict with each other. I liked how there wasn't a massive focus on eating, but I don't think that like kind of looking into why people overeat was really looked into. I feel like the psychology behind it wasn't really like addressed.*  *I'd like to understand why other people might be falling into habits...and*  *understand it a bit more cause personally I can manage my emotions and stuff better when I kind of understand triggers and systems and cycles that can cause things.”* |  | This feedback regarding potential conflict emotions/feelings versus food/eating behaviour and underlying psychological basis not known prior to participant consensus meeting (1 participant made this observation). Research team to consider creating ‘coping tool for difficult feelings’ for future applications of therapy | EXP (Participant)  NCON | No changes made in course of therapy co-development process but registered comment for future therapy development /application | Must do |
| Being taught specific strategies | Would you say it's too much information one session?      Ppt 7: “*No, I think.* | Some strategies had specific benefit:  Ppt 8: “*feel like the breathing exercise really did help calm down on my cravings”.*  AS: *What makes you say that, can explain the process”.*  Ppt 8: “*Well, I feel like it really helped me control my cravings a little bit, so next time it does happen, like when I'm craving, I could just breathe.*  *Use my breathing exercises”.* | Having several specific strategies to work on for each element, clearly highlighted in the manual. It's not clear if strategies are weak or their effectiveness /usefulness is lost on some participants | EXP (Participant) | Specific strategies need to be introduced / appraised on an individual participant basis and based on personal experience and needs based on working with participant to reflects their need / concerns in context of their life | Must do |
| Use of analogies and metaphors | Some young people found the analogies difficult to engage with, but this changed as they assimilated the session content in their lives:  Ppt 7: *There’s a lot of metaphors and analogies, but it did make it easier to understand what she was explaining. I think it has become easier to think about these things as we moved through the sessions.* (session 3) |  |  | EXP | Agreed to continue with use of analogies and metaphors as these were integral to delivery of therapy and basis of supporting materials (i.e, videos) | Must do |
| Goal setting in sessions | It became apparent that young people wanted to talk about specific goals in the sessions:  Ppt3:” *I think we should be more focused on my goals...stuff like that i want to talk about”.* (Session 3) | Ppt 1: “*We also talked about setting a goal for my depression stages as to how we can prevent it from happening... we talked over it and now I'm gonna put as my goal for next week session is to do the table this week through to next week”. (*session 5) |  | EXP | Therapist will ask young people to share their goals in sessions and if they would like to work towards achieving them | Must do |
| Aspect of the intervention (as raised at consensus meetings YP /Professionals) | Negative Comments | Positive comments | Possible changes | Reason for change code | Agreed change | MoScoW  (Must do, Should do, Could do, Would like to do) |
| YP found sessions beneficial towards end of programme and suggested would like more sessions or longer duration | Ppt 2: “*It took a few sessions to kind of get going and then when it was really getting good it was finished.* *So yeah, I think a bit longer might have you know that it might have been quite good in the next 4 sessions”.*  Ppt 4: *“I think that it did take a couple of sessions to get used to what we were like talking about and you know, feeling comfortable to talk to you because I feel like a few more sessions would have been good”.* | Ppt 12: “I *really don't mind. I mean, I have a bunch of free time (now sessions ended). So yes, I just have something to do in that time”.*  Ppt 7: (Mother): “*We thought more sessions as well, didn't we?* *He did quite enjoy them and would have liked it to have carried on a bit longer I think”.* | Provide more sessions as required on individual basis. Not possible within co-development programme for logistical / standardisation reasons. | EXP | No change. However, raised awareness of individual variation in focus and pacing issues within the programme | Would like to do |
| YP questioned spacing of sessions once per week | Ppt 2: “*Maybe more sessions would have been good but if you spread stuff out too much then you kind of lose the kind of train of thought. I guess there's a more of a struggle to connect stuff from different sessions and if I thought something like on Monday and I had to wait all the way until like Saturday to tell you that's already a long time.*  *But if it was like 2 weeks from then I just think it's like too long and there's not really a lot of opportunity to address immediate problems that you're facing”.* | The weekly spacing of session was seen as important as co-developers appreciated the opportunity to build and remember material from week before as they didn’t want to lose the flow.  Ppt 7: “*I think the timing was about right”.*  Ppt 2: “*I don't think having them (sessions) more spread out would have been helpful”.* | Spread out sessions beyond weekly | EXP Participant | NC. Perceived benefit from spreading out sessions and may be detrimental to continuity of therapy for some YP. |  |
| More focus on practice between sessions to increase intrinsic motivation | Young people not keen to have workbook or additional ‘homework’ outside of session as per YP consensus group so unlikely to practice between sessions – although learning from sessions may take time to be assimilated by YP beyond the study period.  If young people are not willing to act differently outside of the sessions, it would suggest an issue with regard to their extent of engagement with the ACT sessions raising the question if additional ‘homework’ should be provided or a workbook to consult outside of the set Aim2Change sessions. | IW: Concerned about homework, but YP do need to be prepared to ‘act’ (e.g. accepting difficult feelings in the service of their values). It's not homework, its facilitating change for them (tweak linguistically or the activities themselves to embed in their life not just about coming back to therapy or clinic appointments)  JW: Generally there was evidence of little practice. They were all kind of quite open about the fact that they didn't do much preparation between the sessions and actually for them. It was kind of a benefit that they just turned up to the session. | Increase expectation setting at the beginning about time in clinic with therapist and time in the week to be implementing/practicing the skills.  & set homework each week – could offer more practice dependent on how this is framed by therapist. | EXP Professional | NC: | Would like to do |
| Clinic ethos / approach may be counter to AIM2Change approach | It was suggested that motivation was extrinsic if engagement was favoured within Coco clinic approach as opposed to engagement with Aim2Change that focuses on eliciting intrinsic motivation. |  | Could ask further questions in the AIM2Change session about whether they’d been to their CEW clinic that week and what issues were raised in consultation?  Consider exit strategy and/or one extra session? CS suggested a take-off and landing (YP already used to clinic structure which is very different). Strengthen the start and return to clinic model. | EXP (Professional) | No Change – but raised awareness of potential of conflict between clinic ethos and Aim2Change approach |  |
| More specific sessions to work on triggers or specific contexts |  | JW: The feedback was that they would like more, and they wanted more specific work on triggers and kind of warning signs for them personally or when their eating behaviour might become difficult. | Flexibility in the content of sessions driven by aspects of young person’s life | EXP | No change – but therapist receptive to YP’s need in session and was also addressed following YP consensus meeting. |  |
| Aim2Change as treatment for dealing with difficult challenges, including attendance at clinic |  | Perceptions of the YP take away from clinic – Advisor might be the key element of the programme to help with this issue  IW: How YP may feel when they are weighed (ACT to understand the world, clinic or family factors are part of that).  Consensus that clinic services are poorly perceived by the YP, but limited time makes it challenging to do more – however Aim2Change intervention has the potential to give YP time to reflect on their experiences. |  | EXP Participant | No change – awareness raised of more global application of Aim2Change and elements of DNA theory such as the Advisor which may serve to elicit constructive reflection on clinic experience |  |
